# The link between bond forfeiture and pretrial release mechanism: The case of Dallas County, Texas

**DOI:** 10.1371/journal.pone.0182772

**Published:** 2017-08-17

**Authors:** Stephen J. Clipper, Robert G. Morris, Amanda Russell-Kaplan

**Affiliations:** 1 Department of Criminology and Criminal Justice, The University of Alabama, Tuscaloosa, Alabama, United States of America; 2 Department of Criminology, University of Texas at Dallas, Richardson, Texas, United States of America; University of Cambridge, UNITED KINGDOM

## Abstract

**Purpose:**

The goal of this study was to evaluate the efficacy of four pretrial jail release mechanisms (i.e., bond types) commonly used during the pretrial phase of the criminal justice process in terms of their ability to discriminate between defendants failing to appear in court (i.e., bond forfeiture). These include attorney bonds, cash bonds, commercial bail bonds, and release via a pretrial services agency.

**Methods:**

A multi-treatment propensity score matching protocol was employed to assess between-release-mechanism differences in the conditional probability of failure to appear/bond forfeiture. Data were culled from archival state justice records comprising all defendants booked into the Dallas County, Texas jail during 2008 (n = 29,416).

**Results:**

The results suggest that defendants released via commercial bail bonds were less likely to experience failure to appear leading to the bond forfeiture process compared to equivalent defendants released via cash, attorney, and pretrial services bonds. This finding held across different offense categories. The study frames these differences within a discussion encompassing procedural variation within and between each release mechanism, thereby setting the stage for further research and dialog regarding potential justice reform.

## Introduction

The pretrial justice system, diversionary programs excluded, in the United States exists for a variety of reasons. These include the need to: a) to physically detain (i.e., jail) defendants to ensure that they stand trial for accused crimes and/or to prevent them from causing further harm and b) to release lower-risk individuals back into society before trial to offset the public burdens associated with jail administration [1]. Approximately sixty-three percent of jail inmates in the US are pretrial detainees [2]. Of those, most will be released prior to their trial via some form of legal bond after a court sets bail (i.e., a dollar amount necessary to facilitate release prior to trial). The bond itself is a legal matter and is intended to ensure that the defendant shall appear in court to stand trial on a future date. Such bonds may be backed by a financial or non-financial guarantee to the court. Financially secured bonds may be backed by cash (or sometimes property) delivered by or on behalf of the defendant or via a privately obtained second-party surety (e.g., a commercial bail bondsman or an attorney). Non-financial bonds may include some form of government funded supervision mechanism (e.g., a pretrial services unit) or simply be the defendant’s promise to appear (i.e., personal recognizance release). While each of these methods of pretrial release is commonplace, little is known regarding their efficacy in terms of preventing failure to appear in court.

Commercial bail bonds, a primary mechanism of pretrial release in the United States, are currently mired in controversy, as they have been at times in the past. In the past few years, increased attention to the pretrial release process, the effects of financially backed release mechanisms on the truly indigent, and potential alternatives have started to call the efficacy and future of financially secured bonds into question. While the aforementioned issues should be and need to be discussed, statements concerning the efficacy of commercial bail bonds are premature as only a few studies have evaluated the ability of commercial bail bonds to achieve the goal of pretrial release: to ensure a defendant released pretrial returns to all subsequent court dates. Of these studies, none have directly compared failure to appear rates for defendants released on commercial bonds to the most commonly applied non-financial alternative, pretrial services release bonds.

The purpose of this study was to provide a balanced empirical evaluation of the performance of varying pretrial release mechanisms in terms of the likelihood of court appearance leading to bond forfeiture for one large urban county in Texas (USA). The availability of a particular type of bond can vary across jurisdictions and can depend on the severity of the accused offense(s), the defendant’s criminal history, and can also be driven by judicial discretion. This reality makes inter-jurisdictional assessments of release mechanisms problematic and caution must be taken in any attempt to make comparisons regarding the performance or utility of one mechanism of pretrial release over another.

Pretrial release itself constitutes the time at which a defendant is released from jail prior to a court hearing where the determination of guilt will be adjudicated. However, there is considerable variation in how a defendant may experience the pretrial justice component of the justice process. In some cases, a defendant undergoes some form of supervised release, or monitoring, while other times he or she may be released without any condition other than a promise to appear in court. Unfortunately, it is this period of time within the criminal justice process that little is known in terms of evidence-based practices and “what works” scholarship. Very few studies, for example, have focused on how different release mechanisms impact defendant outcomes, such as failure-to-appear (FTA) in court, pretrial misconduct, bond forfeiture, recidivism, and so on. It is argued here that understanding the pretrial justice period is critical to enhancing public safety, optimizing the criminal justice process, reducing the burdens of incarceration, and ultimately saving taxpayer dollars.

The practical importance of assessments, such as the present study, is simple: operating a jail is incredibly expensive and jurisdictions are continually challenged by the need to reduce the financial burden of operating jails (i.e., reducing the number jail beds in use), while upholding the constitutional rights of defendants. For example, in Dallas County, Texas, which was home to over 2.4 million inhabitants as of 2014, the cost of jailing an inmate for one night is in excess of fifty (US) dollars. In Dallas County, the average daily jail population generally varies between five and six thousand inmates. Conservatively, and after defendants are released via a pretrial release mechanism, the county spends over $250,000 per day operating the jail, or about $91.3 million annually. Hidden social costs aside, it is easy to see that pretrial release is necessary to operating a criminal justice system; without it, the justice system could not function due to limited jail capacity.

This reality is particularly problematic given that between twenty-five and thirty-three percent of defendants FTA in court post-release from jail to a point at which the bond is forfeited by the court [3] [4], which is costly and burdensome. It is estimated that the procedural cost of a single FTA is roughly $1,700, based on a 2013 dollar value [5]. Beyond the monetary costs associated with FTA, defendants who miss court are not available to receive justice should they be found guilty.

Thus, it makes sense to consider the possibility of modifying justice procedures to maximize the probability of court appearance. There is not, however, a solid understanding of the factors that influence FTA and bond forfeiture or whether the release mechanism, in and of itself, may play a role in the probability of such an occurrence. To complicate matters further, there is considerable inter-jurisdictional variation in how some pretrial release mechanisms are practiced, thereby calling into question studies comparing the utility between different bond types while relying on nationally aggregated data [4], for example. The present study contributes to the body of literature about pretrial release by targeting the first step of the pretrial release process via an assessment of whether distinct pretrial release practices influence FTA in and of themselves in one major metropolitan US jurisdiction and among comparable defendants.

### A brief history of bail as a means of release

While the precise origin of bail is a matter of debate, it is considered one of the oldest criminal justice traditions [6]. The modern state of bail has changed significantly from its English roots (for a chronological development of bail, see [6]). Throughout the use of bail systems, one goal has prevailed: its use as a mechanism to compel a defendant to return to court without the burden of pretrial detention. During this development, a number of bail mechanisms have appeared across the landscape of the American criminal justice system.

Bail has traditionally involved sureties of personal relationships and/or the forfeiture of assets in the event a defendant absconded. This phenomenon evolved in the US during the period of westward expansion [6] when many individuals lacked the personal connections with local contacts that might serve as a meaningful surety [7]. In response to this shortcoming, the commercial bail industry emerged as an alternative to personal sureties. In other words, commercial bondsmen assumed the role of surety-for-hire in a more transient world. Over a period of time, commercial bonds became the primary mechanism of pretrial release, regardless of the defendant’s ability to pay the bond amount [6]. Bail reform efforts in the 1960s and 1980s, however, have changed the nature and function of bail, as well as the mechanisms of release before trial [6]. At present, there are now several forms of release beyond commercial bail bonds. Each of these specific to the present study is briefly outlined below.

### Types of pretrial release mechanisms in Texas

#### Attorney bonds

In Texas, a state licensed attorney may post bond, acting as surety, in counties populated by more than 100,000 residents. For such bonds, the attorney will collect an agreed upon fee, generally a fee ranging between 10 and 20 percent of the bail amount, from the defendant (client) post-release. If the defendant does not appear in court the attorney will be required to remit the bond amount to the county. Note that attorney bonds are unique to Texas.

#### Cash bonds

In most jurisdictions, a defendant may post bail via a cash bond. A cash bond involves the delivery of cash money by the defendant, or on his/her behalf, for the full amount of bail set by the court. If the defendant fails to appear, the bond may be forfeited and the defendant would lose the full amount of the bond. If the defendant appears to stand trial, the money, less court fees, is returned to the defendant. In Dallas County, a defendant who posts a cash bond is released outright with no other formal requirements set forth by the court, other than to appear on the assigned court date.

#### Commercial bonds

A commercial bond is another type of surety bond whereupon a defendant, or his/her friends or family, contacts a for-profit commercial bail bond company, or bond agent, to initiate release for a non-refundable fee. The fee generally ranges between ten and twenty percent of the set bail amount. Commercial bonds are the most commonly used bonds in the country, though not available in seven states and Washington, D.C. at the time of this writing, and represent roughly thirty-three percent of jail releases among all US states [8] [9]. A bail bond agent chooses whether to write the bond and bases this decision on many risk factors, though there is no formally standardized practice within the commercial bond industry. A major factor that is unique to the commercial bail industry involves the use of co-signers, generally more than one, who agree to take responsibility by way of an indemnity should the defendant fail to appear in court. In the event that a defendant absconds, which may be an artifact separate from FTA or result from multiple FTAs, the court may forfeit the bond requiring the bail bond company to remit the full bail amount to the court.

Subsequently, the co-signer(s) would be liable to the bond company for the remainder of the bail amount if the defendant remains unavailable. Interestingly, co-signers may have also had liens put in place on their property in order to obtain the bail bond, rather than delivering cash (e.g., real estate, an auto title, etc.).

In Texas, and in other states that use commercial bonds, the bonding company must be insured by a specially licensed insurance company that acts as surety to the bond company in the case of bond forfeiture. In other words, if the defendant absconds, the bonding company is liable for the full bail amount, with ultimate liability falling to the insurance company. The bond forfeiture process is complex and varies by jurisdiction. In Dallas County, for all release mechanisms assessed here, an initial FTA may or may not result in bond forfeiture. The court may postpone formally changing the bond’s status until an assessment can be made as to the reason for the FTA. There are no set rules on determining what factors will necessarily lead to forfeiture as the criteria are left to the discretion of the court. Should the bond go into forfeiture, the bonding company has a set amount of time to either remit payment or return the defendant to the court. In Dallas County, this period is 180 days for misdemeanors and 270 days for felonies. It is during this window that a bonding company will work with the co-signers to attempt to resolve the forfeiture before calling on a bounty hunter, which is a last-resort, and relatively rare. For more detail on the role of the bounty hunter, see [8], [4]. If this window passes, a hearing is held that requires the bond to be paid in full by the bonding company. At that time, the cosigner(s) becomes civilly liable for the forfeited bond, court costs, and interest.

It is also important to note that should a bonding company believe a client has become at an elevated risk of FTA, or is otherwise not cooperating (i.e., not communicating or not available) may file for an affidavit-to-go-off-bond (ATGOB) with the court. An ATGOB results in a warrant being issued for the defendant before the actual court date so the bonding company can have an opportunity to apprehend the client, remand them to jail, thereby ensuring that the defendant will be present for trial. If an ATGOB does not result in capture and the defendant fails to appear, the bonding company may use the ATGOB as evidence in a bond forfeiture hearing to absolve themselves of having to pay for the forfeited bond.

#### Pretrial services releases

This mechanism occurs as a non-financial release mechanism where defendants are released to the supervision of a publicly sponsored pretrial services department. The goal is that lower-risk indigent defendants can have the opportunity to be released pretrial with the pretrial services department providing non-institutional supervision to the defendants. Pretrial services practice varies widely between jurisdictions. In Dallas County, for example, there are no standard supervision protocols for pretrial services defendants at the present date. Legally speaking, pretrial services releases are commonly classified as a “personal bond,” akin to personal recognizance, but with some supervision and/or conditions of release. It is important to note that defendant’s released via pretrial service bonds have a bond amount set but are not required to provide any financial backing to secure their release. In the event that an individual’s pretrial service bond moves to forfeiture, the court reserves the right to collect on this bond amount.

Washington, D.C. is the quintessential example of a pretrial services agency as the one of the oldest and reportedly most effective examples. The Washington, D.C. pretrial services agency’s self-reported success rate is approximately between 88% and 90% [10]. Interestingly, practitioners and criminal justice officials directly contradict these claims of success criticizing the pretrial services release program and calling for reform. Washington, D.C.’s police chief and mayor have made public statements decrying the state of the District’s criminal justice system of which the pretrial services release agency is a major part (see [11], [12]). In one example, the chief of Washington, D.C.’s police department described a series of crimes committed by a repeat offender that started when a pretrial release GPS monitor ceased to function [11].

It should be noted that the pretrial services release program employed by Dallas County, TX in 2008 was significantly different from other programs in two major ways. First, the pretrial services program run by Dallas County lacked a strong supervision component present in many other programs. During 2008, the pretrial services release department was too understaffed to provide adequate supervision as there were only a handful of officers on staff. Second, Dallas County rarely moved to forfeit pretrial services release bonds, as would occur in other jurisdictions; instead, the county often held these bonds “insufficient” and forego collecting the bond amount. As a result, defendants released via this mechanism in Dallas County, during the study time frame were released on a bond that would be much more accurately compared to an unsecured personal bond or released on recognizance.

#### Release on recognizance

Release on recognizance involves the release of a defendant pretrial based solely on the promise of the defendant to return to court on a specified date. In this release mechanism, defendants who are perceived by the court to be at a low risk of absconding will be released without surety, conditions, supervision, or collateral but solely with the promise of appearing in court. In Dallas County, formal own-recognizance bonds were rarely used for initial book-ins for the time frame under study; consequently, are not explored herein.

### Rationale for financial and non-financial release

In recent decades, there has been considerable controversy over which mechanisms, primarily financial or non-financial, should be utilized in the justice system. This debate originates with a perception of unfairness toward the indigent in the financial release system. Research has suggested that that poorer defendants may be more likely to be detained compared to defendants with financial resources (see [13], [14], [15]), though many such studies are dated. The inequity in the bail system is important to acknowledge because of potential for detainment for no reason other than a lack of financial resources.

Additionally, research has identified that some defendants held pretrial are more likely to plead guilty than those who are released [16]. Based on a sample of nearly 800 defendants in New Jersey, Sacks and Ackerman [16] suggested that this occurs more frequently with low-level charges and may occur so that the defendant can be released from jail rather than to remain, regardless of guilt is factual. The authors also suggested that those detained pretrial may often receive harsher sentences, if convicted, though this may not be the case system-wide [16]. It is problematic that the inability, for whatever reason, to acquire a financially based release could lead an increased likelihood of plea-bargaining.

Countering this position, proponents of financial release argue that financial release mechanisms are necessary due to their ability to inhibit absconding by increasing the collateral on a defendant’s behalf, thus enhancing encouragement for standing trial. Often, commercial bonds will include co-signers to this aim, as discussed. This perception has even permeated the courts as seen in the decision of *Hinton v. United States* [17] in which the rationale of the court was that indigent defendants released via financial mechanisms posted by family and friends will have more incentive to return to court. This debate continues today, mostly in the political arena [6]. Additionally, some commercial bail agents report using payment plans, rather than a flat fee, for securing release which may result in very low amounts of cash needed to secure the release of a defendant at the time of release, questioning the reality of unavailable release for indigent defendants in some jurisdictions.

Despite polarization of ideas on which philosophy of pretrial release brings the most utility or the most virtue, two aspects are clear: 1) there are truly indigent defendants who remain in jail for no reason other than financial limitations; and, 2) the present justice system could not function without forms of financial release. Indeed, there is limited empirical research of quality that addresses the efficacy of pretrial release mechanisms on FTA and forfeiture rates. Helland and Tabarrok published one such study, relying on a counterfactual research design [4]. In an analysis of defendants from seventy-five of the largest counties in the US, there was evidence that defendants released on a surety bond were twenty-eight percent less likely to fail to appear compared to personal recognizance and eighteen percent less likely compared to deposit bond [4]. Deposit bonds are akin to cash bonds except the court will collect a fraction of the full bond amount in lieu of the full cash amount. Furthermore, absconders released via commercial bonds had the lowest percent of absconding periods over one year compared to personal recognizance, deposit bond, and cash bond. This study provided evidence of the effect of commercial bond mechanisms in being more successful than other mechanisms; however, it was limited by the fact that aggregated national data were used that did not account for variation in the availability of any particular release mechanism and variation in pretrial release conditions between jurisdictions. In truth, some types of release may not be available for the same defendant in one jurisdiction over another, making comparisons inappropriate. Furthermore, some release mechanisms under the same title vary considerably form one jurisdiction to another (e.g., pretrial services).

There is also empirical evidence supporting the effectiveness of pretrial services in ensuring court appearance. For example, a multi-city experiment conducted in 1985 utilizing random assignment explored the effect of pretrial services release for defendants previously denied bail [18]. The findings suggested that pretrial services release can be an effective release tool for appropriately screened defendants. More specifically, the authors found that an average of eighty-six percent of such defendants returned to all court hearings and eighty-eight percent were not arrested prior to their trial [18]. Also worthy of note, all defendants included in the study were previously unable to be released before their trial. As a result, this study provides evidence of the possibility for an effective non-financially based program to achieve the goals of pretrial release (e.g., improving release rates), while additionally preserving community safety and compelling the defendant to return to court. Interestingly, the authors also estimated the jail space saved by the pretrial services programs, in this case, approximately 256 beds per year across the three sites [18]. However, it is critical to recall that pretrial services agencies vary considerably in their availability and execution.

Finally, in FTA discussion and scholarship, it is important to note that all FTAs are not necessarily nefarious. Helland and Tabarrok discussed this in their review of bail mechanisms [4]. The authors noted that defendants sometimes fail to appear because they fell ill or simply forgot about the obligation, etc. Some evidence suggests that simple reminders can improve FTA rates. One recent study that collected data from 14 counties in Nebraska found that defendants who were randomly selected to receive varying forms of a mailed reminder about appearance were more likely to appear than those who did not [19]. Additionally, reminders that included details about the potential penalties resulting from FTA (rather than the appearance alone) were found to enhance appearance rates even more. However, the study did not account for variation in the form of release mechanism so it is unclear whether the combination of a mailer and the facets of a particular release mechanism drove the effect. For example, a defendant who received a particular reminder and was released via a very active pretrial services program with supervision requirements is quite different than another defendant who also received the same reminder protocol but was released on recognizance.

Notwithstanding the previously mentioned studies of pretrial release mechanisms, there are a number of questions left unanswered. Specifically, it is unclear whether different forms of pretrial release can impact the probability of court appearance among similarly situated defendants. The current study addresses this gap in the literature specific to comparable defendants released via commercial bonds, attorney bonds, cash bonds, and pretrial services bonds for one of the largest local criminal justice systems in the US: Dallas County, Texas. In order to achieve a more nuanced understanding, the current study evaluates the efficacy of these release mechanisms in all defendants followed by stratifications of offense seriousness and offense type. In order to explore this relationship, the study evaluates felony and misdemeanor defendants as well as defendants charged with drug, DWI, and larceny offenses.

## Methods

### Data

This study was approved by the institutional review board at the University of Texas at Dallas (approval number: MR15-353). Data for the present study were culled from archived jail, court, and arrest records made available by Dallas County, Texas. These data represent all official criminal and court records on file involving every adult defendant booked into and release from the county jail during 2008.

These data included detailed historical information regarding defendants’ criminal histories (through 2011; provided by the Texas Department of Public Safety to account for arrests on file outside of Dallas County), socio-demographic data, biological information, calendar information for every file on record from book-in to disposition (e.g., celerity, time in jail, etc.), and other important information tied to each specific release from jail (e.g., through which release mechanism did the release transpire). As the goal of this study was to isolate the effect of respective pretrial release mechanisms among like defendants, a focal arrest was established to mark the beginning of the criminal justice process for a particular criminal event (or set of events falling under one arrest). Here, the focal arrest corresponds to the first book-in for each defendant occurring during 2008 for an offense(s) in which no previous arrest had occurred. Thus, by design, each defendant’s progress through the criminal justice process, for the set of charges corresponding to the focal arrest, along with any subsequent criminal justice record, was tracked from the time of initial arrest through 2011. This strategy was critical for isolating any effect of a release mechanism because the mechanism should be tethered only to the release to which it was responsible. This approach standardized progress through the justice system for all defendants under study.

To clarify, each defendant in these data appear only once and correspond to the first arrest of the 2008 calendar year. Any data relating to specific offenses occurring before 2008 or new offenses after the focal arrest were excluded except where necessary to identify a history of FTA or future recidivism. In these two scenarios, these data were linked to the focal arrest. This was done to ensure that FTAs were specifically linked to the focal arrest and not the result of any charge occurring before or after the focal arrest.

The pretrial release mechanism by which a defendant is released can be considered the front line of reentry back into society and is the focus of the present study. Here, an attempt was made to isolate the effect of particular release mechanisms on subsequent FTA leading to bond forfeiture, net of other effects, and between similarly situated defendants.

### Measurement

#### Outcome variable

The final outcome variable for the study was whether a defendant failed to appear in court following pretrial release from jail for the focal arrest (1 = failed to appear; 0 = did not fail to appear). For attorney, cash, and commercial bonds, FTA was indicated by a judgment *nisi* issued by the court, which marks the beginning of the bond forfeiture process and a bench warrant being issued for the defendant who has failed to appear. For pretrial services release in Dallas County, however, FTA does not automatically result in a judgment *nisi* or a bench warrant. Rather, when a defendant fails to appear on a pretrial services bond, the bond is held *insufficient*. Through communication with county officials, it was determined that pretrial services bonds held insufficient were the best possible indicator of FTA for that release mechanism in Dallas County.

#### Treatment variables

The present study estimates the causal effects of multiple treatments through a counterfactual modeling strategy–discussed below. In all, four treatments each representing a different pretrial release mechanism were explored and compared to one another. These include pretrial release via an attorney bond, a cash bond, a commercial bond, or a pretrial services bond (though here, such rarely included supervision). Each of these pretrial mechanisms was defined previously. Personal recognizance bonds were not included due to a very low representation for initial releases (less than 1 percent of defendants).

#### Covariates

The Dallas County data included numerous legal and extra-legal characteristics specific to each defendant, many of which have, until now, never been available in an empirical assessment of FTA. These variables, described here, are listed below. The following extralegal covariates were used to estimate a propensity score for each treatment type comparison: gender (female = 1; male = 0), age, z-score centered age-squared, race coded as a categorical variable (Black, White, or Hispanic; White = reference category), indigence (indigent = 1; not indigent = 0); marital status (married = 1; not married = 0), mental illness history (present = 1; not present = 0), medical problems (present = 1; not present = 0), and birthplace (born in the US = 1; foreign born = 0). Additionally, several legal factors were also used to estimate FTA propensity. These included the number of prior arrests on record, the calendar year of the initial arrest, prior book-in in the Dallas County jail (yes = 1; no = 0), FTA history (at least one previous FTA = 1; no FTA history = 0), the number of days spent in jail for the focal arrest, celerity (number of days between the offense date and the book-in date; log transformed to adjust for skew), and the type of offenses (charges) involved with the focal arrest. For the latter, these included categorical indicators for a drug-related, family violence, homicide, sexual assault, robbery, assault, burglary, larceny, fraud, auto theft, obstruction of justice, weapon-related charge, and/or DWI/DUI charge. Here, a defendant may have varying charge types associated with the focal arrest (e.g., robbery along with a weapon-related charge) and this variation is modeled explicitly here with the reference category being a charge not involving any of the types for which were controlled.

- Extralegal
  - Gender (female = 1; male = 0)
  - Age
  - Z-score centered age-squared
  - Race coded as a categorical variable (Black, White, or Hispanic; White = reference category)
  - Indigence (indigent = 1; not indigent = 0)
  - Marital status (married = 1; not married = 0)
  - Mental illness history (present = 1; not present = 0)
  - Medical problems (present = 1; not present = 0)
  - Birthplace (born in the US = 1; foreign born = 0)
- Legal Factors
  - Number of prior arrests on record
  - Calendar year of the initial arrest
  - Prior book-in in the Dallas County jail (yes = 1; no = 0)
  - FTA history (at least one previous FTA = 1; no FTA history = 0)
  - Number of days spent in jail for the focal arrest
  - Celerity (number of days between the offense date and the book-in date; log transformed to adjust for skew)
- Current Charge (yes = 1; no = 0)
  - Drug-related
  - Family violence
  - Homicide
  - Sexual assault
  - Robbery
  - Assault
  - Burglary
  - Larceny
  - Fraud
  - Auto theft
  - Obstruction of justice
  - Weapon-related charge
  - DWI/DUI charge

### Relying on matched samples to isolate pretrial release mechanism effects

The effect of one mechanism over another was assessed via a quasi-experimental design (counterfactual) known as propensity score matching (PSM). PSM provides a useful means for transparently establishing equivalence between a treatment and a matched control group, for the purpose of assessing mean differences on an outcome variable, here whether FTA occurred. In short, both treatment and control groups are matched on a vector of conditions or attributes, ideally balancing the probability of receiving the treatment (i.e., release mechanism) or not, analogous to an experimental research design, but not a replacement for an experiment. The PSM method in which matched controls who are statistically indistinguishable from those who received the treatment are identified is a random process that can deem contextual differences between treated versus control groups ignorable [20], [21], [22]. PSM is well positioned to study differences in effectiveness between pretrial release mechanisms in terms of FTA for a situation where randomized trials are not feasible, but where processes specific to assignment to one mechanism over another can be statistically modeled using observed archival data and variables that likely confound the raw probability of a defendant being released by one mechanism over another [23]. Indeed, such methods are common within the criminological literature [24], [25].

Through this approach, the assumption of “conditional independence” must be satisfied. This occurs only when (1) a reasonable degree of certainty can be empirically established and, (2) that any underlying processes driving the likelihood of assignment to one treatment over another is ignorable once the propensity for treatment is adequately controlled via temporally preceding features correlated to receiving the treatment itself. By way of this assumption, propensity scores can be used to match “treated” defendants to similarly situated defendants who were equally likely to have received that same “treatment” (i.e., release mechanism), but did not. Propensity scores are thus defined as “the conditional probability of assignment to a particular treatment given a vector of observed covariates” [20] (p.41).

An important aspect of the current application of PSM lies in the fact that it involves an assessment of multiple treatments simultaneously. Though relatively rare, such an approach has been used in similar studies (e.g., see [4]; [26], [27]. In this study, the likelihood of FTA was explored across multiple mechanisms of pretrial release for defendants at an equivalent stage in the criminal justice process. Since there were multiple treatments under study, comparisons were made from one release-type to another for every possible combination of treatments, respectively. The goal was to end up with a conditioned estimate of the “treatment effect” for one release type over all other types separately. This was the difference in the mean probability for defendants failing to appear between two specific release mechanisms. Again, these comparisons were based on statistically matched defendants who were equally likely to have received the treatment. In the end, comparisons were made not between all defendants released by way of a particular method, but only between statistically matched pairs (i.e., apples-to-apples).

The matching procedure itself is carried out by identifying one or more equally likely untreated counterparts to every treated case in terms of estimated propensity score. For the analysis at hand, the 1-to-1 nearest neighbor algorithm was employed, without replacement [28]. Further, a condition for matching one case to another lies in the distance between a treated case and an untreated case on the score itself, know as the “caliper.” For present purposes, the caliper was conservatively set at 0.001.

Within the context of exploring differences in the effect of various pretrial release mechanisms on the probability of court appearance, PSM offers a transparent method for which problems surrounding the counterfactual are addressed. This protocol accounts for selection bias that otherwise may influence whether a defendant is released via one mechanism over another. Through a counterfactual design such as this, the experimental design is approximated with regard to the fundamental of equal probability of selection to the treatment, or control group, otherwise known as “balance.”

The first step in the process of creating comparable groups of defendants for each release mechanism combination was to estimate the propensity scores on a vector of covariates, all of which were outlined in the preceding section, by way of probit regression. Plainly, these propensity scores are the estimated probabilities of being released via one mechanism over another for the offense of record. As noted, each alternative comparison was assessed resulting in a total of 12 probit regression models for six different pairings of release mechanisms (e.g., commercial vs. cash, cash vs. commercial, pretrial services vs. commercial, commercial vs. pretrial services, and so on) and for each stratification (i.e., all defendants, felonies, misdemeanors, drug offenses, DWI offenses, and larceny offenses). These stratifications were included to understand the presence and nature of any variations in efficacy stemming from offense type. Inclusion of drug offenses, DWI offenses, and larceny offenses was the result of the frequency of these charges which lent well to matching. Other specific offense types were available (i.e., homicide) but it occurred too infrequently to be match effectively.

**Fig 1:**
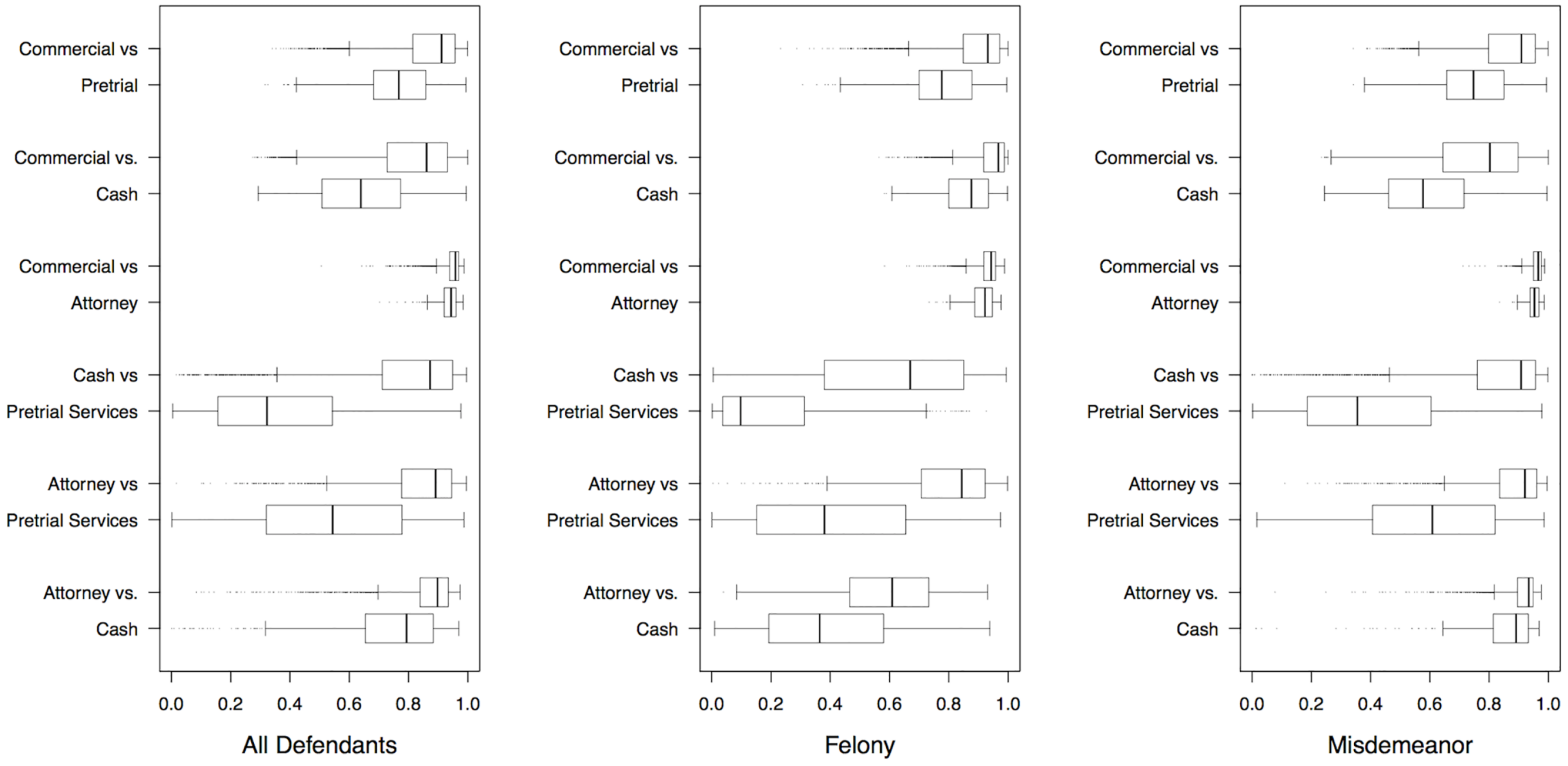
Propensity score overlap between groupings, by stratification. Box-whisker plots illustrating overlap between groupings on the propensity for being released via a particular mechanism.

Two models were assessed for each mechanism comparison within each stratification in order to ensure differences were not artifacts of the PSM algorithm. Helland and Tabarrok [4] summarize the efficacy of this process when matching across multiple treatments. Once these propensity scores were estimated, the overlap in propensity scores between treatment and matched control groups for each analysis were visually examined via box-whisker plots to assess the potential for finding matches for treated cases. Fig 1 is a visual depiction of the propensities to be released via the top mechanism for both mechanisms. For example, the upper left of the figure presents the distribution of propensities for defendants that were released via commercial bonds or pretrial services to be released via commercial bonds. The visual interpretation of these box-whisker plots is to ensure that there is overlap between these mechanisms in the propensity for release. In the absence of sufficient overlap, there would not be sufficient cases available to match. This would result in few if any cases being matched across mechanism, especially when using a caliper of 0.001. The selection of box-whisker plots is provided to demonstrate the presence of an overlap across mechanisms and offense types. In each case, there was adequate overlap for matched cases to be identified.

The following step involved an assessment of covariate balance across each comparison group post-estimation of propensity scores between treated cases and their matched controls. Independent samples *t*-tests were carried out for all covariates between treated versus untreated and treated versus matched controls with the goal of demonstrating that the matching procedure effectively removed bias between those treated and the controls as a result of the matching procedure. Due to the volume of comparisons for the present analysis, the *t*-test results specific to balancing are omitted herein, however, the matching procedure effectively removed all covariate differences in each stratification and grouping post-matching (i.e., *t* was less than 1.96 in each instance).

Upon establishing balance, tests for differences between the final outcomes were calculated via *t*-tests for each matched group comparison. Sensitivity of the results was assessed via the Mantel and Haenszel [29] test statistic (MH; commonly referred to as *mhbounds*) as it applies to assessing the impact of unmeasured covariates in propensity score estimation for binary outcomes. The MH statistic represents the magnitude, in the form of an odds ratio, that an unmeasured covariate would need to be represented by in order to potentially confound a treatment effect–more detail on sensitivity in PSM can be found in DiPrete and Gangl [30]. Estimated treatment effects and sensitivity analyses for each comparison grouping are discussed in the following section.

## Results

This study explored the efficacy of four pretrial release mechanisms on FTA by assessing differences in the probability of FTA between a series of pairings of Dallas County, Texas defendants released via either of one type of mechanism to another, until all possible comparisons were established. These four mechanisms included attorney bonds, cash bonds, commercial bonds, and pretrial services bonds. Rates of FTA were first compared between mechanism pairings for all defendants. Crime types were then broken down to parse out efficacy for felony and misdemeanor offenses, as well as offense-specific categories. Comparisons were created with each mechanism acting as a treatment and a control in order to address differences in FTA rates resulting from disparate ranges of effects. Table A in S1 Appendix presents the summary statistics for all defendants released via each of the four mechanisms and across each strata assessed.

Table 1 presents conditional differences between release types resulting from the PSM analysis. These PSM findings are presented to illustrate the conditional differences in FTA rates between those treated and their matched controls for all releases, felonies, and misdemeanors, respectively. On the diagonal of these tables are the unadjusted FTA rates for each release type. These raw statistics are presented for reference and are suggestive of commercial bonds being the more successful in terms of reducing the probability of FTA compared to any other release type studied. The off-diagonal statistics are the conditional mean difference in FTA rates (i.e., the treatment effects) between those released via a particular mechanism versus a single alternative. This is identified by the left-hand column, compared to a single alternative, identified by the top row of the table. Note that the number range discussed (if statistically significant) reflects the estimated difference for matching based on an inverted treatment outcomes (e.g., commercial vs. attorney and attorney vs. commercial), which is important to consider given that matching may not be equivalent for the inverse of a treatment condition [27].

**Table 1:** Charge levels: Average treatment effects: Failure to appear (Unconditioned rates on the diagonal).

Bond Type	Attorney	Cash	Commercial	Pretrial Services
**All Defendants**				
Attorney	[0.34]	0.01	0.12*	-0.05
Cash	0.00	[0.30]	0.10*	-0.02
Commercial	-0.12*	-0.10*	[0.23]	-0.15*
Pretrial Services	0.02	0.02	0.12*	[0.39]
**Felony Defendants**				
Attorney	[0.28]	-0.05	0.11*	-0.01
Cash	0.06	[0.31]	0.17*	0.03
Commercial	-0.11*	-0.15*	[0.17]	-0.09*
Pretrial Services	0.00	-0.05	0.11*	[0.25]
**Misdemeanor Defendants**				
Attorney	[0.38]	0.01	0.15*	0.07
Cash	-0.02	[0.31]	0.07*	-0.05*
Commercial	-0.10*	-0.08*	[0.29]	-0.12*
Pretrial Services	-0.06	0.02	0.11*	[0.40]

*p < 0.05

When assessing differences using data from all defendants, only the comparisons involving commercial bonds were found to be statistically significant. In this study, commercial bond defendants tended to fail to appear at a significantly lower rate compared to each alternative. This difference was largest between commercial bonds and pretrial services bonds, which ranged between 0.12 and 0.15. The smallest reduction was between commercial and cash release mechanisms. Commercial bonds produced a FTA rate 0.10 lower than cash bonds and a rate of 0.12 lower than attorney bonds. Comparisons between other bond mechanisms did not produce statistically significant differences. These general findings suggest that commercial bonds may be more effective at compelling a defendant to return to court appearances than other mechanisms, when equivalent defendants are compared. Analyses then focused specifically on felonies and misdemeanors, respectively.

For felonies and misdemeanors, a similar pattern of findings emerged. Commercial bond defendants were less likely to fail to appear compared to alternative mechanisms, respectively. This trend again continued for comparisons using misdemeanor offenses with one exception. Results indicated a statistically significant difference between cash bonds and pretrial services bonds with cash bond defendants being more likely to appear in court. These analyses provide additional evidence that those released via commercial bonds tended to have lower FTA rates compared to other mechanisms across both felony and misdemeanor charges.

To supplement the above analyses, comparisons were made on FTA rates for specific charge/offense types where data were amply available. These results are presented in Table 2. Specifically, these involved defendants charged with drug offenses, driving while intoxicated, and larceny (theft), respectively. In general, defendants released on commercial bonds again tended to FTA at lower rates compared to other mechanisms for these crime specific comparisons. Regarding those defendants charged with a drug related crime, those released via commercial bonds were significantly more likely to appear in court for adjudication. No other between-mechanism comparisons were found to be significantly different.

**Table 2:** Specific crime types: Average treatment effects: Failure to appear (Unconditioned rates on the diagonal).

Bond Type	Attorney	Cash	Commercial	Pretrial Services
**Drug Defendants**				
Attorney	[0.32]	0.08	0.11*	-0.12
Cash	0.06	[0.35]	0.13*	-0.05
Commercial	-0.10*	-0.11*	[0.25]	-0.10*
Pretrial Services	0.04	0.06	0.11*	[0.34]
**DWI Defendants**				
Attorney	[0.35]	-0.04	0.12*	-0.07
Cash	0.01	[0.29]	0.08*	-0.06
Commercial	-0.12	-0.07*	[0.24]	-0.13*
Pretrial Services	0.12	0.05	0.17*	[0.39]
**Larceny Defendants**				
Attorney	[0.41]	0.03	0.15	0.02
Cash	-0.11	[0.33]	0.08*	0.00
Commercial	-0.06	-0.10*	[0.27]	-0.11*
Pretrial Services	-0.05	0.12	0.12*	[0.36]

*p < 0.05

Comparisons analyzing defendants charged with driving while intoxicated produced evidence of similar reductions from commercial bonds when compared to cash bonds and pretrial services bonds. However, conditional differences between commercial bonds and attorney bonds for driving while intoxicated were not significant.

The differences in FTA rates between mechanisms for defendants charged with larceny were similar to driving while intoxicated defendants. There was a statistically significant difference between commercial bonds when compared to cash bonds and pretrial services bonds. In both cases, defendants released on a commercial bond had lower rates of FTA compared to the other mechanism. The comparison between attorney bonds and commercial bonds produced partial support for a difference. There was a significant difference when attorney bonds were matched to commercial bonds with attorney bonds acting as the treatment and commercial bonds acting as the control, though the reverse was not as such, lending only partial support to this comparison.

Overall, these findings tend to suggest that when comparing similar defendants in equivalent contexts, those released on a commercial bail bond for initial release from jail pending trial are consistently less likely to fail to appear resulting in bond forfeiture. This difference was found to be present across varying strata of offenses. However, it should be noted that these analyses may be sensitive to unmeasured effects, as is the case in most counterfactual studies. Sensitivity analysis indicated that unmeasured variables with modest odds ratios could have impacted matching, however, the bulk of the findings were robust to sensitivity assessments (i.e., bounds statistics). An overview of the sensitivity of the above results is discussed in the following section.

In all counterfactual research designs, it is important to take into consideration the potential for hidden bias (i.e., that which may stem from unmeasured variables). In the present case, data were limited to official records, thus leaving some potentially important features associated with FTA left unmeasured such as personal beliefs, situational information, and court room workgroup variables. One approach to assessing the potential for an impact from such unmeasured bias, if indeed it had been available, is to estimate bounds statistics (see [28], [30], [31], and [32]. Following the protocol outlined by Rosenbaum [32], *gamma* coefficients (MH) were estimated for each matching procedure, resulting in dozens of estimates in this case. These results are presented in Table 3. For each statistically significant finding, *gamma* ranged between 1.4 and 4.0 between groupings, suggesting that depending on the particular matching procedure, an omitted feature would need have a conditional effect, in terms of an odds ratio, in excess of 1.4 to offset the finding of any of the statistically significant difference tests presented in this study (see Table 3). On average, *gamma* was 2.4, suggesting less of a concern for the influence of omitted variables and that these results are relatively robust to hidden bias.

**Table 3:** The effects of pretrial release mechanism on failure to appear (FTA) for four pretrial release mechanisms in Dallas County, Texas.

Treat Mech.	Control Mech.	FTA (Treat)	FTA (Control)	FTA Diff.	% Diff.	t	Γ
All							
Attorney	Commercial	0.35	0.23	0.12	0.34	4.93	2.1
Commercial	Attorney	0.23	0.35	-0.12	-0.52	-5.05	2.2
Cash	Commercial	0.30	0.20	0.10	0.33	10.04	1.8
Commercial	Cash	0.20	0.30	-0.10	-0.50	-9.70	1.8
Commercial	Pretrial	0.22	0.37	-0.15	-0.68	-11.05	2.2
Pretrial	Commercial	0.37	0.25	0.12	0.32	9.03	1.9
Felony							
Attorney	Commercial	0.28	0.17	0.11	0.39	5.88	2.6
Commercial	Attorney	0.17	0.28	-0.11	-0.65	-3.65	2.7
Cash	Commercial	0.31	0.14	0.17	0.55	5.62	4.0
Commercial	Cash	0.17	0.32	-0.15	-0.88	-4.62	3.1
Commercial	Pretrial	0.17	0.26	-0.09	-0.53	-3.66	2.0
Pretrial	Commercial	0.26	0.15	0.11	0.42	5.13	2.6
Misdemeanor							
Attorney	Commercial	0.38	0.23	0.15	0.39	4.65	2.6
Commercial	Attorney	0.28	0.38	-0.10	-0.36	-3.02	2.0
Cash	Commercial	0.31	0.24	0.07	0.23	5.87	1.5
Commercial	Cash	0.23	0.31	-0.08	-0.35	-7.37	1.7
Cash	Pretrial	0.33	0.38	-0.05	-0.15	-2.06	1.5
Commercial	Pretrial	0.28	0.40	-0.12	-0.43	-6.96	1.9
Pretrial	Commercial	0.40	0.29	0.11	0.28	6.63	1.9
Drug							
Attorney	Commercial	0.33	0.22	0.11	0.33	2.25	2.6
Commercial	Attorney	0.23	0.33	-0.10	-0.43	-2.00	2.4
Cash	Commercial	0.35	0.22	0.13	0.37	3.88	2.8
Commercial	Cash	0.24	0.35	-0.11	-0.46	-3.38	2.2
Commercial	Pretrial	0.24	0.34	-0.10	-0.42	-4.15	2.0
Pretrial	Commercial	0.34	0.23	0.11	0.32	4.66	2.1
DWI							
Cash	Commercial	0.31	0.23	0.08	0.26	4.10	1.7
Commercial	Cash	0.24	0.31	-0.07	-0.29	-3.64	1.6
Commercial	Pretrial	0.25	0.38	-0.13	-0.52	-3.09	2.7
Pretrial	Commercial	0.38	0.21	0.17	0.45	4.00	3.3
Larceny							
Cash	Commercial	0.34	0.26	0.08	0.24	2.25	1.9
Commercial	Cash	0.24	0.34	-0.10	-0.42	-2.78	2.1
Commercial	Pretrial	0.25	0.36	-0.11	-0.44	-4.17	2.1
Pretrial	Commercial	0.36	0.24	0.12	0.33	4.40	2.2

note: all *p* < 0.05

## Discussion and conclusion

The present study contributes to the literature by offering a comparative assessment of the utility of varying pretrial defendant release mechanisms in terms of FTA in court, leading to bond forfeiture. What is unique about the present study is that it is the first to assess these effects between similarly situated defendants using comprehensive archival records from Dallas County, Texas. The focus on a single jurisdiction is important as to avoid cross-jurisdiction comparisons where justice systems vary considerably and impact the likelihood and availability of any given release mechanism. In general, the findings are robust in suggesting that comparable defendants released via a commercial bond tend to be more likely to appear in court as compared to attorney, cash, and pretrial services bonds. This finding held across many strata of offense categories, however, these effects should be taken in context.

All pretrial release mechanisms are not equal in the resources available to compel a defendant to return to court. Helland and Tabarrok [4] outline these differences, which cover many domains. First, the commercial bail industry is permitted to require co-signers and collateral. This practice, however, is not permitted in other pretrial release mechanisms. Second, commercial bond agencies regularly contact defendants to remind them of court appearances. These reminders can increase the likelihood of appearing for court [19]. Third, commercial bond agencies collect detailed information on the defendant that can be used to help locate him/her in the event the defendant absconds and can always refer to the co-signers for information. Bail bond agencies gather information on the defendant’s family, employment, and residence, for example [4]. Finally, in the event a defendant absconds, commercial bond agencies have extraordinary tools available to return the defendant to court including arrest, imprisonment, and transportation across state lines [33]. These differences (e.g., the use of bounty hunters) demonstrate the unequal field of promoting court appearance. None of the mechanisms evaluated here are provided all of these tools to encourage defendants appear for trial.

Commercial bond agencies can even petition the court to remove them as the bonding agent in cases where the defendants are not cooperating with the terms of their bail. This is done with an *affidavit-to-go-off-bond* (ATGOB). In the event that the court accepts the ATGOB, the liability of the bonding agency is removed and an arrest warrant is put out for the defendant [34].

Despite the lower FTA rate of commercial bail, each mechanism plays an important role pretrial release. This is particularly true for pretrial services bonds as this service allows indigent defendants to be released pretrial in the absence of financial collateral. Financial restrictions should not preclude an individual from pretrial release, especially when pretrial detention affects criminal justice system processing. Nevertheless, the criminal justice system needs to balance the competing aims of equitable pretrial release while ensuring a defendant’s appearance in court.

Considering that over sixty percent of jail inmates in the US are being held pretrial [2], it is paramount that the criminal justice system has mechanisms to relieve this burden without wasting tax dollars. It is also important, however, for defendants to appear in court. FTA in court not only permits the defendant to escape judgment but also has an inherent expense to the criminal justice system. Block and Twist estimate that each FTA cost the criminal justice system approximately $1,273 or closer to $1,700 in 2009 [5]. In Dallas, this equates to an approximated cost of $12,466,100 for the 7,333 FTAs for 2009 alone, or nearly $35,000 per day. Considering the substantial cost associated with FTA in a single county court, there is considerable fiscal incentive to reduce FTA in addition to the pursuit of justice.

While relatively robust, the current study is not without limitations. First, these analyses stem from a single jurisdiction in a single state. Second, while this study was able to determine the efficacy of bail mechanisms across various offenses, the low frequency of some violent crimes, such as sexual assault, prevented further granularity in the analysis. Third, release on own recognizance bonds were rarely applied in Dallas County during the data collection and thus were prevented from inclusion in the study. It should be noted that a pretrial services release in Dallas County, during the study period, was akin to release on recognizance bonds.

Finally, accurately measuring FTA and bond forfeiture is challenging. The complexities associated with capturing FTA are outlined in Dallas County Bail Bond Task Force’s report [34]. At the most basic level, a FTA would occur when a defendant does not show up to a scheduled court proceeding requiring the defendant’s presence. The bailiff would record a FTA if the defendant does not appear within a reasonable time after being called. In Dallas County, defendants and attorneys are allowed to appear at-will. This increases the difficulty of identifying FTA and challenges the prosecutor to be cognizant of appearances and the necessity to request bond forfeiture. Furthermore, the judges and court staff maintain information of FTA, instead of the bailiff, as part of court records, but there is no requirement for collecting or maintaining such data.

In Texas, the court decides when to forfeit a bond and there is no mandated guideline or criteria for forfeiture *per se*. For example, a defendant may fail to appear many times prior to the court actually forfeiting the bond. In another court in the same jurisdiction, a judge may have a strict forfeiture policy where a single FTA results in forfeiture. A possible solution for this discrepancy would be to require the court to record appearance information in addition to bond forfeiture.

Despite these limitations, this study increases the knowledge of the efficacy of different pretrial release mechanisms, especially when parsing out the effect by offense type. Here, commercial bonds consistently produce the lowest rate of FTA and forfeiture compared to other mechanisms. Effectiveness of commercial bonds aside, it is important to acknowledge each mechanism has its merits, especially with indigent defendants.

It should be noted that the authors are not advocating for or against any mechanisms in particular. The current study is an analysis of pretrial release options available in Dallas County, Texas in 2008. As such, the results may not be generalizable to other periods in time or other jurisdictions. Instead, it is important to acknowledge that all of these mechanisms coexist as independent components in a complex pretrial release process. It would be difficult to predict how the pretrial release system would function after any major change to these mechanisms. Simply ending the use of one or more mechanisms in favor of another could have unforeseen consequences resulting in drastically different efficacy. Until more is understood about pretrial release, failure to appear, each of these mechanisms individually, and how they operate in the system together, caution is warranted prior to any policy decisions. This is especially true considering the pretrial services release department operating in Dallas County, Texas during 2008 lacked many standard components of pretrial services agencies.

Future research should focus similar analyses on alternative jurisdictions. There is significant potential for between jurisdiction variation based on laws, court practices, individual discretion, and program quality. Future research should also look at the conditions imposed by pretrial release mechanisms to attempt to identify the most significant predictors of successful appearance. This knowledge could increase the use of evidence-based practices in all pretrial release mechanisms.

## Supporting information

S1 Appendix Descriptive statistics by release type. (DOCX)
