# Expression of Concern: The link between bond forfeiture and pretrial release mechanism: The case of Dallas County, Texas

**DOI:** 10.1371/journal.pone.0236275

**Published:** 2020-07-13

**Authors:** The PLOS ONE Editors

Following publication of this article [1], concerns have been raised regarding the availability of the underlying data.

Prior to publication of this article, the corresponding author declared that the Institutional Review Board of University of Texas at Dallas restricted public sharing of the underlying data set given this included potentially identifying information. In accordance with the PLOS Data Availability Policy, and with editorial agreement, the Data Availability Statement for the article provided contact details for the institutional officials to whom requests for the data set could be addressed.

It has been raised to the attention of the *PLOS ONE* Editors that the Center for Crime and Justice Studies, University of Texas at Dallas, where the data was reportedly archived, has closed, and the former directors of the Center are no longer at the University. The authors are no longer affiliated to the University of Texas at Dallas. The *PLOS ONE* Editors have followed up with the authors and with the institution, but have been unable to obtain the data or confirmation that it has been retained. The authors have indicated that the records used to compile the study data set are held by the Criminal Justice Department of Dallas County and the Texas Department of Criminal Justice. Defendant records were matched across several unique identifiers used by various agencies.

Additionally, the following information is missing from the Competing Interests statement: RGM has previously been contracted by the American Bail Coalition for expert testimony. There are no patents, products in development or marketed products associated with this research to declare. The other authors declare that they have no competing interests.

The *PLOS ONE* Editors issue this Expression of Concern to inform readers that the underlying data set used in this study is not available at the time of publication of this notice.
