# Supplementary material for: The link between bond forfeiture and pretrial release mechanism: The case of Dallas County, Texas
Source: PLoS One. 2017 Aug 17;12(8):e0182772. doi: 10.1371/journal.pone.0182772 (PMC5560530; doi:10.1371/journal.pone.0182772)
Supplement: S1 Appendix — (DOCX) [file pone.0182772.s001.docx]

| **S1Appendix. Table A. Descriptive Statistics by Release Type** | | | | | | | | | | | | | | | |
| --- | --- | --- | --- | --- | --- | --- | --- | --- | --- | --- | --- | --- | --- | --- | --- |
|  |  | **Attorney: Obs = 806** | | | |  | **Cash: Obs = 4,377** | | | |  | **Commercial: Obs = 15,321** | | | |
| **Variable** | | Mean | SD | Min | Max |  | Mean | SD | Min | Max |  | Mean | SD | Min | Max |
|  | Male | 0.81 | 0.40 | 0 | 1 |  | 0.80 | 0.40 | 0 | 1 |  | 0.77 | 0.42 | 0 | 1 |
|  | Age | 31.67 | 10.78 | 17 | 75 |  | 32.19 | 10.77 | 17 | 86 |  | 30.75 | 10.68 | 16 | 82 |
|  | Age^2^ (z-score) | 1.04 | 1.77 | 0 | 17.48 |  | 1.05 | 1.85 | 0 | 27.24 |  | 1.01 | 1.59 | 0 | 23.44 |
|  | Black | 0.30 | 0.46 | 0 | 1 |  | 0.16 | 0.37 | 0 | 1 |  | 0.44 | 0.50 | 0 | 1 |
|  | Hispanic | 0.39 | 0.49 | 0 | 1 |  | 0.40 | 0.49 | 0 | 1 |  | 0.25 | 0.43 | 0 | 1 |
|  | Indigence | 0.58 | 0.49 | 0 | 1 |  | 0.44 | 0.50 | 0 | 1 |  | 0.67 | 0.47 | 0 | 1 |
|  | Medical Problems | 0.07 | 0.25 | 0 | 1 |  | 0.06 | 0.24 | 0 | 1 |  | 0.08 | 0.27 | 0 | 1 |
|  | Mental Health | 0.13 | 0.34 | 0 | 1 |  | 0.05 | 0.22 | 0 | 1 |  | 0.13 | 0.34 | 0 | 1 |
|  | US Born | 0.93 | 0.26 | 0 | 1 |  | 0.90 | 0.30 | 0 | 1 |  | 0.95 | 0.22 | 0 | 1 |
|  | Married | 0.24 | 0.43 | 0 | 1 |  | 0.23 | 0.42 | 0 | 1 |  | 0.20 | 0.40 | 0 | 1 |
|  | Year of First DPS | 2001.08 | 8.70 | 1959 | 2008 |  | 2003.91 | 7.29 | 1953 | 2008 |  | 2001.07 | 8.53 | 1940 | 2008 |
|  | Total Arrests before 2008 | 2.66 | 3.89 | 0 | 25 |  | 1.23 | 2.42 | 0 | 33 |  | 2.62 | 3.59 | 0 | 33 |
|  | FTA before 2008 | 0.06 | 0.24 | 0 | 1 |  | 0.03 | 0.17 | 0 | 1 |  | 0.07 | 0.26 | 0 | 1 |
|  | Prior Jail | 0.42 | 0.49 | 0 | 1 |  | 0.25 | 0.43 | 0 | 1 |  | 0.44 | 0.50 | 0 | 1 |
|  | Current Offense |  |  |  |  |  |  |  |  |  |  |  |  |  |  |
|  | Felony | 0.48 | 0.49 | 0 | 1 |  | 0.08 | 0.27 | 0 | 1 |  | 0.35 | 0.48 | 0 | 1 |
|  | Celerity (ln) | 0.87 | 1.33 | 0 | 6.27 |  | 0.67 | 1.25 | 0 | 5.38 |  | 0.84 | 1.31 | 0 | 7.14 |
|  | Days in Jail | 5.49 | 14.42 | 0 | 198 |  | 1.52 | 9.58 | 0 | 481 |  | 3.73 | 14.82 | 0 | 751 |
|  | Drug | 0.22 | 0.42 | 0 | 1 |  | 0.09 | 0.29 | 0 | 1 |  | 0.20 | 0.40 | 0 | 1 |
|  | Family Violence | 0.10 | 0.30 | 0 | 1 |  | 0.09 | 0.29 | 0 | 1 |  | 0.12 | 0.33 | 0 | 1 |
|  | Sexual Assault | 0.02 | 0.14 | 0 | 1 |  | 0.00 | 0.05 | 0 | 1 |  | 0.01 | 0.08 | 0 | 1 |
|  | Robbery | 0.02 | 0.14 | 0 | 1 |  | 0.00 | 0.04 | 0 | 1 |  | 0.01 | 0.11 | 0 | 1 |
|  | Aggravated Assault | 0.17 | 0.38 | 0 | 1 |  | 0.11 | 0.31 | 0 | 1 |  | 0.17 | 0.38 | 0 | 1 |
|  | Burglary | 0.04 | 0.21 | 0 | 1 |  | 0.01 | 0.10 | 0 | 1 |  | 0.04 | 0.19 | 0 | 1 |
|  | Larceny | 0.09 | 0.28 | 0 | 1 |  | 0.09 | 0.28 | 0 | 1 |  | 0.12 | 0.32 | 0 | 1 |
|  | Fraud | 0.02 | 0.13 | 0 | 1 |  | 0.01 | 0.11 | 0 | 1 |  | 0.03 | 0.16 | 0 | 1 |
|  | Auto Theft | 0.00 | 0.06 | 0 | 1 |  | 0.00 | 0.04 | 0 | 1 |  | 0.01 | 0.08 | 0 | 1 |
|  | Obstructing Justice | 0.10 | 0.30 | 0 | 1 |  | 0.08 | 0.28 | 0 | 1 |  | 0.11 | 0.31 | 0 | 1 |
|  | Weapon | 0.03 | 0.16 | 0 | 1 |  | 0.02 | 0.15 | 0 | 1 |  | 0.03 | 0.17 | 0 | 1 |
|  | DWI | 0.15 | 0.35 | 0 | 1 |  | 0.34 | 0.47 | 0 | 1 |  | 0.12 | 0.33 | 0 | 1 |

Continued from above.

|  |  | **Pretrial Serv. Obs = 2,480** | | | |  | **Full Sample: Obs = 29,416** | | | |
| --- | --- | --- | --- | --- | --- | --- | --- | --- | --- | --- |
| **Variable** | | Mean | SD | Min | Max |  | Mean | SD | Min | Max |
|  | Male | 0.66 | 0.47 | 0 | 1 |  | 0.77 | 0.42 | 0 | 1 |
|  | Age | 29.61 | 11.80 | 17 | 78 |  | 30.73 | 10.59 | 16 | 86 |
|  | Age^2^ (z-score) | 1.25 | 1.67 | 0 | 19.93 |  | 1.00 | 1.58 | 0 | 27.24 |
|  | Black | 0.52 | 0.50 | 0 | 1 |  | 0.40 | 0.49 | 0 | 1 |
|  | Hispanic | 0.21 | 0.41 | 0 | 1 |  | 0.27 | 0.44 | 0 | 1 |
|  | Indigence | 0.86 | 0.35 | 0 | 1 |  | 0.65 | 0.48 | 0 | 1 |
|  | Medical Problems | 0.07 | 0.26 | 0 | 1 |  | 0.07 | 0.26 | 0 | 1 |
|  | Mental Health | 0.16 | 0.37 | 0 | 1 |  | 0.13 | 0.34 | 0 | 1 |
|  | US Born | 0.95 | 0.21 | 0 | 1 |  | 0.94 | 0.24 | 0 | 1 |
|  | Married | 0.11 | 0.32 | 0 | 1 |  | 0.20 | 0.24 | 0 | 1 |
|  | Year of First DPS | 2001.89 | 8.87 | 1951 | 2008 |  | 2001.43 | 8.34 | 1940 | 2008 |
|  | Total Arrests before 2008 | 1.68 | 2.61 | 0 | 29 |  | 2.55 | 3.60 | 0 | 37 |
|  | FTA before 2008 | 0.03 | 0.16 | 0 | 1 |  | 0.08 | 0.27 | 0 | 1 |
|  | Prior Jail | 0.39 | 0.49 | 0 | 1 |  | 0.43 | 0.49 | 0 | 1 |
|  | Current Offense |  |  |  |  |  |  |  |  |  |
|  | Felony | 0.29 | 0.45 | 0 | 1 |  | 0.29 | 0.45 | 0 | 1 |
|  | Celerity (ln) | 0.61 | 1.17 | 0 | 6.91 |  | 0.94 | 1.38 | 0 | 7.14 |
|  | Days in Jail | 2.30 | 3.43 | 0 | 95 |  | 6.08 | 26.41 | 0 | 1396 |
|  | Drug | 0.29 | 0.46 | 0 | 1 |  | 0.19 | 0.39 | 0 | 1 |
|  | Family Violence | 0.00 | 0.00 | 0 | 0 |  | 0.09 | 0.29 | 0 | 1 |
|  | Sexual Assault | 0.00 | 0.02 | 0 | 1 |  | 0.01 | 0.07 | 0 | 1 |
|  | Robbery | 0.00 | 0.00 | 0 | 0 |  | 0.01 | 0.10 | 0 | 1 |
|  | Aggravated Assault | 0.00 | 0.03 | 0 | 1 |  | 0.13 | 0.33 | 0 | 1 |
|  | Burglary | 0.03 | 0.16 | 0 | 1 |  | 0.03 | 0.17 | 0 | 1 |
|  | Larceny | 0.26 | 0.44 | 0 | 1 |  | 0.12 | 0.32 | 0 | 1 |
|  | Fraud | 0.05 | 0.22 | 0 | 1 |  | 0.02 | 0.16 | 0 | 1 |
|  | Auto Theft | 0.02 | 0.12 | 0 | 1 |  | 0.01 | 0.06 | 0 | 1 |
|  | Obstructing Justice | 0.10 | 0.31 | 0 | 1 |  | 0.10 | 0.31 | 0 | 1 |
|  | Weapon | 0.00 | 0.00 | 0 | 0 |  | 0.02 | 0.15 | 0 | 1 |
|  | DWI | 0.09 | 0.29 | 0 | 1 |  | 0.15 | 0.36 | 0 | 1 |
